# Testosterone affects language areas of the adult human brain

**DOI:** 10.1002/hbm.23133

**Published:** 2016-02-15

**Authors:** Andreas Hahn, Georg S. Kranz, Ronald Sladky, Ulrike Kaufmann, Sebastian Ganger, Allan Hummer, Rene Seiger, Marie Spies, Thomas Vanicek, Dietmar Winkler, Siegfried Kasper, Christian Windischberger, Dick F. Swaab, Rupert Lanzenberger

**Affiliations:** ^1^ Department of Psychiatry and Psychotherapy Medical University of Vienna Austria; ^2^ MR Center of Excellence, Center for Medical Physics and Biomedical Engineering, Medical University of Vienna Austria; ^3^ Department of Obstetrics and Gynecology Medical University of Vienna Austria; ^4^ Netherlands Institute for Neuroscience, Institute of the Royal Netherlands Academy of Arts and Sciences Amsterdam the Netherlands

**Keywords:** testosterone, language, neuroplasticity, voxel‐based morphometry, probabilistic tractography, functional connectivity

## Abstract

Although the sex steroid hormone testosterone is integrally involved in the development of language processing, ethical considerations mostly limit investigations to single hormone administrations. To circumvent this issue we assessed the influence of continuous high‐dose hormone application in adult female‐to‐male transsexuals. Subjects underwent magnetic resonance imaging before and after 4 weeks of testosterone treatment, with each scan including structural, diffusion weighted and functional imaging. Voxel‐based morphometry analysis showed decreased gray matter volume with increasing levels of bioavailable testosterone exclusively in Broca's and Wernicke's areas. Particularly, this may link known sex differences in language performance to the influence of testosterone on relevant brain regions. Using probabilistic tractography, we further observed that longitudinal changes in testosterone negatively predicted changes in mean diffusivity of the corresponding structural connection passing through the extreme capsule. Considering a related increase in myelin staining in rodents, this potentially reflects a strengthening of the fiber tract particularly involved in language comprehension. Finally, functional images at resting‐state were evaluated, showing increased functional connectivity between the two brain regions with increasing testosterone levels. These findings suggest testosterone‐dependent neuroplastic adaptations in adulthood within language‐specific brain regions and connections. Importantly, deteriorations in gray matter volume seem to be compensated by enhancement of corresponding structural and functional connectivity. *Hum Brain Mapp 37:1738–1748, 2016*. © **2016 Wiley Periodicals, Inc**.

**Abbreviations**
AF: Arcuate fasciculus
DTI: Diffusion tensor imaging
DWI: Diffusion weighted images
EmC: Extreme capsule
FA: Fractional anisotropy
FAI: Free androgen index
FWE: Family wise error rate
GMV: Gray matter volume
MPRAGE: Magnetization prepared rapid gradient echo
MRI: Magnetic resonance imaging
SHBG: Sex hormone‐binding globulin
VBM: Voxel‐based morphometry

## INTRODUCTION

Testosterone exhibits a considerable influence on human behavior through modulation of brain structure and function [Hofer et al., 2013]. This includes, but is not limited to, bargaining and dominant behavior [Eisenegger et al., 2010], neuronal activation in response to visuo‐spatial processing and threat [Goetz et al., 2014] as well as size and number of neurons [Bao and Swaab, 2011]. Moreover, it plays a particular role in language function. Increased fetal testosterone predicts smaller vocabulary [Lutchmaya et al., 2002], resulting in better language performance of girls compared to boys [Hollier et al., 2013], and it affects the processing and lateralization of language function in infants. Accordingly, previous studies reported negative associations between testosterone and gray matter volume (GMV) in children [Lombardo et al., 2012] and adults [Witte et al., 2010] within the left superior temporal gyrus (Wernicke's area) and the inferior frontal gyrus (Broca's region), reflecting the two major brain areas involved in language processing.

As a main limitation, the majority of human studies can only assess the cross‐sectional effects of testosterone or the response to a single dose of hormones due to ethical, methodological and practical reasons [Lombardo et al., 2012]. In this context, investigation of transsexual subjects offers the unique opportunity to study the influence of high‐dose long‐term hormone application onto the living human brain in healthy adults. They exhibit strong and persistent cross‐gender identification, often seeking for hormonal treatment and sex reassignment surgery [Bao and Swaab, 2011], which in turn enables investigation of specific hormone effects avoiding otherwise confounding aspects of simple sex differences. In line with the above mentioned influence of testosterone on behavior, several studies in female‐to‐male (FtM) transsexuals reported deteriorating effects on language performance associated with the administration of androgens, in contrast to an increase in spatial ability performance in the same group [Gooren and Giltay, 2008; Van Goozen et al., 1995].

To investigate specific effects of long‐term testosterone administration on human brain structure and function, 18 FtM transsexuals underwent magnetic resonance imaging (MRI) before and after four weeks of hormone intervention to circumvent the mentioned issues of cross‐sectional investigations. As FtM subjects receive continuous high‐dose testosterone, we were particularly interested in effects of total serum testosterone (*T*_total_), bioavailable testosterone (*T*_bio_) and free androgen index (FAI). Our first objective was to identify gray matter brain regions that change in accordance with alterations in testosterone. T1‐weighted structural MRIs were subject to a voxel‐based morphometry (VBM) analysis using an optimized processing pipeline for longitudinal assessment [Zatorre et al., 2012]. Second, we aimed to investigate the influence of testosterone on the corresponding white matter fiber tracts connecting the gray matter regions identified above. Here, diffusion weighted images (DWI) were used for probabilistic tractography. Similar to the investigation of structural connections, we finally evaluated functional connectivity between the altered gray matter regions by use of resting‐state functional MRI data obtained at 7 Tesla.

Based on previous investigations, we expected that increases in testosterone levels will be associated with decreases in GMV in brain regions relevant for language processing [Lombardo et al., 2012; Witte et al., 2010]. Similarly, decreases in structural and functional connectivity were expected as increased testosterone has been shown to decrease white matter integrity [Peper et al., 2015] and functional connectivity in adolescence [Peters et al., 2015].

Although our primary hypotheses concerned the investigation of direct associations between changes in testosterone levels and changes in imaging parameters, we also investigated more general effects of the treatment period as compared to a control group of female volunteers not receiving hormonal treatment.

## METHODS

### Subjects

Eighteen female‐to‐male transsexual subjects were included in this study (mean age ± sd = 27.3 ± 6.4 years). Diagnosis of gender identity disorder was assessed according to the criteria listed in the Diagnostic and Statistical Manual of Mental Disorders, 4^th^ edition (DSM‐IV) by an experienced psychiatrist at the screening visit. Briefly, this was diagnosed as a strong and persistent (> 6 months) cross‐gender identification and discomfort with the current sex, causing clinically significant distress or impairment in social, occupational or other areas of functioning. Of note, the term gender identity disorder has changed to gender dysphoria in DSM‐5 with a focus on dysphoria as a clinical problem (rather than the identity per se) and to avoid stigmatization. Transsexual subjects did not fulfil criteria for current comorbidities but three reported history of a specific phobia (*n* = 1), anorexia (*n* = 1) or bulimia nervosa (*n* = 1). All patients reported subjective feelings to belong to the other gender before or at puberty and they wanted sex reassignment independent of study participation. To assess general effects of treatment, a control group of 16 healthy female subjects was also included (24.6 ± 5.2 years). All subjects underwent standard medical examinations with routine laboratory blood and pregnancy tests, electrocardiography and assessment of general physical and neurological status. Exclusion criteria were presence or history of any severe physical or neurological disorders, substance abuse, prior intake of psychotropic medication and hormones (including contraceptives), pregnancy and contraindications to MRI scanning. All subjects provided written informed consent after detailed explanation of the study protocol and they were reimbursed for study participation. This study was approved by the Ethics Committee of the Medical University of Vienna and procedures were performed according to the Declaration of Helsinki.

### Study Design and Treatment

All transsexual subjects underwent MRI before and at least four weeks after (34.9 ± 7.1 days, mean ± sd) start of hormone treatment. Female control subjects did not receive hormone treatment between the two MRI scans. Hormone treatment was carried out according to established protocols of the Department of Obstetrics and Gynecology, Unit for Gender Identity Disorder at the Medical University of Vienna [Kranz et al., 2015]. FtM transsexuals were administered either 1000 mg testosterone undecanoate every 12 weeks (Nebido® 250 mg/ml, 4 ml vial in castor oil, intramuscular, *n* = 15) or 50 mg testosterone daily (Testogel® 50mg/5g bag, transdermal, *n* = 3). If menstruation persisted, subjects additionally received 10‐15 mg lynestrenol (Orgametril® 5 mg, oral) or 75 μg desogestrel (Cerazette® 75 μg, oral) daily. Blood samples were drawn prior to MRI scanning at each visit. Plasma levels of total serum testosterone (*T*_total_), sex hormone‐binding globulin (SHBG) and bioavailable testosterone (*T*_bio_) were determined at the Department of Laboratory Medicine, Medical University of Vienna (http://www.kimcl.at). The FAI was computed as FAI = *T*_total_/SHBG*100.

### Magnetic Resonance Imaging (MRI)

All structural MRI measurements were obtained on a 3 Tesla scanner (Siemens Trio, Erlangen, Germany) using a 32‐channel head coil [Hahn et al., 2015a]. Structural images were acquired with a T1‐weighted magnetization prepared rapid gradient echo (MPRAGE) sequence (TE/TR = 4.2/2,300 ms, image resolution = 1.1 × 1 × 1 mm, 160 sagittal slices). DWI were acquired in the same scanning session with a single‐shot diffusion‐weighted echo planar imaging sequence (TE/TR = 83/8,700 ms, flip angle = 90°, image resolution 1.64 mm isotropic, 70 axial slices, *b*‐value = 800 s/mm^2^) in 30 diffusion‐encoding directions and one non‐diffusion weighted b0‐image. On the same day, functional MRI measurements were obtained at 7 Tesla (Siemens Magnetom) using a 32‐channel head coil [Hahn et al., 2015b]. Briefly, a single‐shot echo‐planar imaging sequence was employed (TE/TR = 23/1,400 ms, image resolution = 1.5 × 1.5 × 2 mm plus 1 mm slice gap, 32 axial slices). Subjects completed a 6 min resting‐state functional MRI scan with instructons to stay awake, keep eyes open (fixed on a crosshair) and to not focus on anything in particular (“let thoughts come and go freely”).

### Voxel‐Based Morphometry (VBM)

T1‐weighted images were segmented and spatially normalized using the DARTEL (Diffeomorphic Anatomical Registration using Exponentiated Lie algebra) module [Ashburner, 2007] as implemented in the VBM8 toolbox (http://dbm.neuro.uni-jena.de/vbm/) for SPM8 (http://www.fil.ion.ucl.ac.uk/spm/) [Kraus et al., 2014]. Default parameters were used but the normalization process was optimized for longitudinal VBM analyses [Hagemann et al., 2011; Lanzenberger et al., 2013] as recommended by the VBM8 toolbox developers. For each subject, the two structural scans were realigned to their mean image and corrected for field inhomogeneities. The mean image was then spatially normalized to MNI‐space and the transformation matrix was applied to the individual scans. The resulting gray matter segments were converted to GMV by multiplication with the Jacobian determinants obtained during the spatial normalization procedure [Ashburner and Friston, 2009] to adjust for effects of non‐linear spatial normalization. Finally, GMV maps were again realigned to their mean and smoothed with an 8mm Gaussian kernel [Kraus et al., 2014].

### Diffusion Tensor Imaging (DTI) Analysis

Processing of DWIs and tractography were carried out with the FMRIB software library (FSL v5.0.5, http://fsl.fmrib.ox.ac.uk/fsl/fslwiki/) using default parameters unless specified otherwise [Hahn et al., 2015a]. This included adjustment for eddy currents as well as removal of the skull and non‐brain tissue with the brain extraction tool. The tensor was fitted with a weighted least squares model, yielding estimates of fractional anisotropy (FA) as well as axial, radial and mean diffusivity (AD, RD, MD). Tractography was done with FSL's Diffusion Toolbox in individual space [Behrens et al., 2003]. Local diffusion parameters were computed with 5,000 sample streamlines and 2 fiber directions per voxel [Behrens et al., 2007], which enables modeling of crossing fibers and tracking of non‐dominant pathways. Probabilistic tractography was carried out between brain regions identified in the VBM analysis, namely for the arcuate fasciculus (AF) and the extreme capsule pathway (EmC) [Saur et al., 2008]. To provide optimal seed regions for tracography [Croxson et al., 2005; Floel et al., 2009; Saur et al., 2008], VBM results were binarized at *P* < 0.05 family wise error rate (FWE)‐corrected at cluster level (following *P* < 0.001 uncorrected voxel level), smoothed with an 8 mm Gaussian kernel and thresholded at 50%. As the obtained regions comprised the left middle superior temporal and inferior frontal gyri (see results), additional coronally oriented waypoint and/or exclusion masks were created at the level of the central sulcus (*y* = −20 mm) [Fernandez‐Miranda et al., 2012] and the extreme capsule perpendicular to the claustrum (*y* = 2 mm) [Saur et al., 2008]. Hence, to reconstruct the AF only those tracts were kept, which pass through the two cortical and the central sulcus ROIs but not the extreme capsule mask. Conversely, for the EmC, only those tracts were included, which pass through the two cortical and the extreme capsule masks but not the central sulcus ROI. To register the ROIs to individual space, inverse transformation matrices of spatial normalization (obtained during segmentation of T1‐weighted images) and coregistration (FA image to GMV maps) were applied to the ROIs [Croxson et al., 2005]. To remove spurious results, voxels exhibiting the lowest 10% of sample streamlines or areas with FA < 0.2 were removed in individually reconstructed tracts [Croxson et al., 2005; Floel et al., 2009], as probabilistic tractography represents the robustness to model tracts in the presence of noise. Furthermore, as tractography results may depend on the order of seed and target regions, tracts were computed in both “directions” (A‐>B and B‐>A) and combined afterwards. Finally, mean values of FA, AD, RD and MD were extracted from the obtained tracts [Jones et al., 2006]. For visualization purposes (but not for quantitative assessment) individually reconstructed tracts were spatially normalized to MNI‐space using the above mentioned forward transformation matrices and averaged across all subjects [Saur et al., 2008].

### Resting‐State Functional MRI

Functional images were preprocessed in SPM8 as described previously [Hahn et al., 2015b]. This included slice timing correction (reference = middle slice), motion correction (reference = mean image), spatial normalization to MNI‐space and spatial smoothing (8 × 8 × 8 mm Gaussian kernel). Spatial normalization was carried out using the deformation fields obtained during segmentation of the T1‐weigted images (after coregistration to structural images). Resting‐state data were analyzed in Matlab R2011a (The Mathworks, Natick, MA). Here, band‐pass filtering (0.009 < *f* < 0.08Hz, implemented by discrete cosine transformations) and removal of potentially confounding signals (motion parameters, white matter and ventricular signals) was carried out in a single regression analysis to avoid the introduction of artifacts [Hallquist et al., 2013]. Furthermore, we did not correct for global signal as this may alter the interpretation of resting‐state fMRI results [Saad et al., 2012]. Functional connectivity was computed between the same seed regions used for tractography analysis, namely the left middle superior temporal and inferior frontal gyri. Average time courses of the two regions were extracted and cross‐correlation coefficient was calculated, followed by *z*‐transformation.

### Statistical Analysis

Unless stated otherwise, all results are corrected for multiple comparisons as described below. To investigate the associations between changes in testosterone and changes in GMV, voxel‐wise linear regression analysis was computed in SPM8 at *P* < 0.05 corrected for multiple comparisons using the family wise error rate (FWE) at cluster level following *P* < 0.001 voxel level. Here, the difference GMV map between scan 1 and scan 2 was computed, serving as dependent variable. On the other hand, the difference in testosterone levels (*T*_bio_, FAI and *T*_total_) between scan 1 and scan 2 was used as independent variable. Likewise, correlations were calculated between changes in DTI metrics and changes in testosterone levels between the two scans in Matlab. Here, correction for multiple comparisons was carried out using the Bonferroni procedure for two tracts * three testosterone metrics, hence, *P* < 0.05/6 = 0.0083. Associations between changes in functional connectivity and changes in testosterone levels between scan 1 and scan 2 were calculated in the same manner. To exclude influence of potentially confounding variables, all calculations were also carried out with corrections for baseline values of *T*_bio_, age, total intracranial volume as well as GMV in peak voxels, tract diffusivities or functional connectivity for gray, white matter and resting‐state analyses, respectively. To test general effects of the treatment period (secondary hypotheses), a repeated measures ANOVA was computed, testing the interaction effect between factors group (transsexuals vs. controls) and time (first vs. second MRI scan). As stated above, differences in GMV were computed in SPM8, whereas changes in structural and functional connectivity were assessed in Matlab.

## RESULTS

### Treatment

Time between start of the hormone treatment and the second MRI scan was 34.9 ± 7.1 days. Measurements at 7 Tesla were obtained on the same day as the corresponding 3 Tesla scans. As expected, testosterone levels significantly changed after the treatment period. In detail, *T*_total_ increased from 0.36 ± 0.17 ng/mL at the first scan to 3.76 ± 2.07 ng/mL at the second scan (*t* = 7.3, *P* < 0.001), *T*_bio_ increased from 0.11 ± 0.07 ng/mL to 1.81 ± 1.39 ng/mL (*t* = 5.3, *P* < 0.001) and the FAI increased from 0.72 ± 0.50 to 13.35 ± 12.45 (*t* = 4.4, *P* < 0.001).

### Effects of Testosterone on Gray Matter Volume

**Figure 1:**
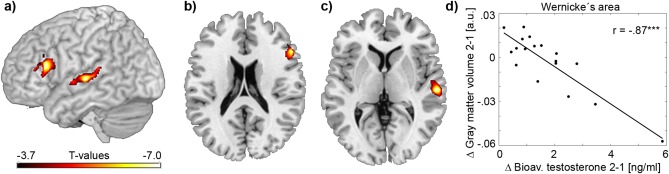
Associations between bioavailable testosterone and GMV. Voxel‐wise linear regression analysis was conducted between changes in bioavailable testosterone and changes in GMV between MRI scan 1 and scan 2. Results are superimposed on a 3D surface of the brain (**a**) and on axial slices at *z* = 20 mm (**b**) and *z* = 4 mm (**c**), *P* < 0.05 FWE‐corrected for multiple comparisons at cluster level. Significant negative associations were observed in the left inferior frontal gyrus (b, peak voxel *t* = −7.55, *x*/*y*/*z* = −54/23/15 mm, mean cluster *t* = −4.63, cluster size = 1985 mm^3^) and the left middle superior temporal gyrus (c, peak *t* = −7.08, *x*/*y*/*z* = −60/‐21/1 mm, mean cluster *t* = −4.60, cluster size = 2396 mm^3^). The scatterplot exemplarily represents the association at the peak voxel in the left middle superior temporal gyrus (**d**). *r*: Pearson's correlation coefficient. ***P < 0.001 **uncorrected**. Axial slices are in radiological convention (left is right). [Color figure can be viewed in the online issue, which is available at http://wileyonlinelibrary.com.]

Regression analysis revealed that changes in *T*_bio_ were predictive of changes in GMV within the left inferior frontal gyrus (Broca's area, *r* = −0.88, mean cluster *r* = −0.76, Fig. 1a,b) and the left middle superior temporal gyrus (Wernicke's area, *r* = −0.87, mean cluster *r* = −0.75, Fig. 1a,c,d). Analysis without the potential outlier subject exhibiting highest changes in *T*_bio_ slightly decreased the correlations for both Broca's (*r* = −0.69) and Wernicke's areas (*r* = −0.74). Similar effects were observed for FAI (*r* = −0.86 and *r* = −0.84 for Broca's and Wernicke's areas, respectively) but not for *T*_total_, which indicates specific effects of testosterone available for tissue uptake. The results were not changed after additional correction for potentially confounding baseline values of *T*_bio_ or regional GMV, total intracranial volume or age (all *r* = −0.84…−0.91).

### Effects of Testosterone on Structural Connections

**Figure 2:**
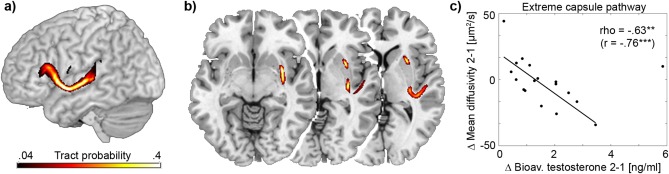
Associations between bioavailable testosterone and white matter diffusivity. Individual tract reconstruction was carried out using probabilistic tractography for the white matter fiber pathway of the extreme capsule. Average tract probabilities across all subjects are superimposed on a 3D surface of the brain (**a**) and on axial slices at *z*=‐7/‐2/3 mm (**b**). The scatterplot represents the association of changes in bioavailable testosterone and changes in mean diffusivity between MRI scan 1 and scan 2 (**c**). Since one individual was a clear outlier, Spearman's rho was calculated for all subjects, whereas Pearson's *r* and the regression line were computed without the outlier (denoted in brackets). **P < 0.01, ***P < 0.001. Axial slices are in radiological convention (left is right). [Color figure can be viewed in the online issue, which is available at http://wileyonlinelibrary.com.]

As the gray matter analysis specifically identified language regions, the second experiment aimed to evaluate the influence of testosterone on corresponding structural connections between Broca's and Wernicke's areas. These include the two major language tracts of the AF dorsally [Catani and Mesulam, 2008] and the ventral pathway through the extreme capsule (EmC) [Saur et al., 2008]. Investigating the microstructural characteristics of white matter tracts between the two MRI scans showed negative associations between changes in EmC mean diffusivity and changes in *T*_bio_ (rho = −0.63, Fig. 2), except for a single subject (*r* = −0.76 without outlier). Similar results were obtained for changes in FAI (*r* = −0.67) and *T*_total_ (*r* = −0.76). The obtained associations in mean diffusivity were driven by corresponding changes in both axial (*r* = −0.62) and radial diffusivity (*r* = −0.67), hence, cancelling out correlations with changes in FA (*r* = 0.34, *P* > 0.05 uncorrected). The observed effect sizes were similar to those reported earlier. That is 50µm^2^/s change in MD after testosterone correspond to 7% change from baseline, whereas [Rametti et al., 2012] showed changes in FA between 5% and 8% for different tracts. Interestingly, the predictive value was specific for the EmC as no associations were observed for the AF independent of the testosterone or diffusivity metrics used (all rho < 0.37, *r* < 0.47, *P* > 0.05 uncorrected). Again, the findings were not changed when correcting for potentially confounding values of *T*_bio_ or tract mean diffusivity at baseline, total intracranial volume or age (*r* = −0.71…‐0.76 and *r* = −0.36…−0.55 for EmC and AF).

### Effects of Testosterone on Functional Connectivity

**Figure 3:**
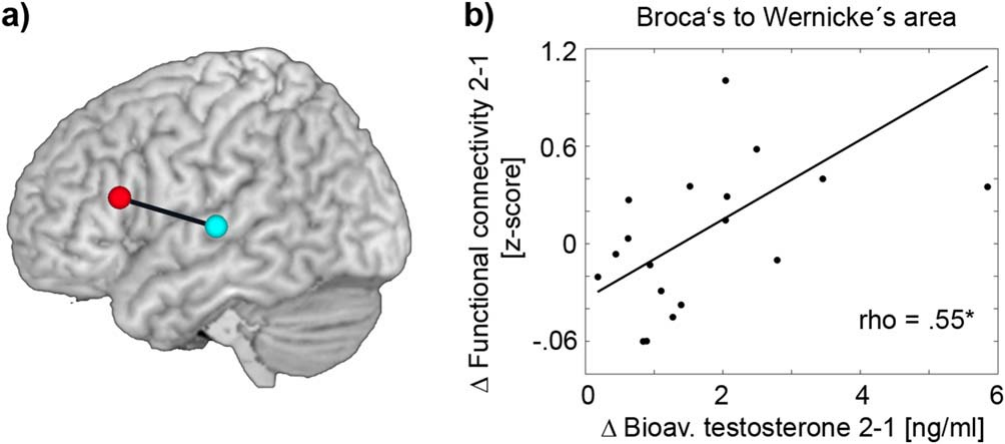
Association between bioavailable testosterone and functional connectivity. Resting‐state functional MRI was used to compute functional connectivity between left inferior frontal gyrus and left superior temporal gyrus (i.e., clusters identified in the VBM analysis, figure 1). Cluster centers are schematically superimposed on a 3D surface of the brain (**a**). The scatterplot represents the association of changes in bioavailable testosterone and changes in functional connectivity between MRI scan 1 and scan 2 (**b**). Since one individual was an outlier Spearman's rho was computed and the regression line plotted without this subject. *P < 0.05. [Color figure can be viewed in the online issue, which is available at http://wileyonlinelibrary.com.]

The third assessment was carried out to investigate if the influence of testosterone on the functional connectivity between Broca's and Wernicke's areas is in accordance with that of testosterone on structural connections. Regression analysis showed a positive association for changes in functional connectivity between the two scans and changes in *T*_bio_ (rho = 0.55, Fig. 3). Similar results were obtained for changes in *T*_total_ (rho = 0.6), but not for FAI. Correction for potentially confounding values of *T*_bio_ or functional connectivity at baseline, total intracranial volume or age did not change this finding (rho = 0.5…0.58).

### General Effects of the Treatment Period

We further tested changes between the two MRI scans in transsexuals as compared to healthy controls (secondary hypothesis). For GMV significant interaction effects of group * time were limited to few occipital regions (left middle *t* = 5.3, *k* = 465mm^3^; right superior *t* = 5.12, *k* = 1,225 mm^3^; right lingual *t* = 4.39, *k* = 789mm^3^) and the cerebellum (left *t* = 4.76, *k* = 1,063mm^3^, all *P* < 0.05 FWE‐corrected cluster level). There were no significant interaction effects within the AF or EmC pathways for any of the diffusion metrics (all *P* > 0.05). Furthermore, there was no significant interaction for functional connectivity between Broca's and Wernicke's areas (*P* > 0.05).

Furthermore, no significant correlations were observed between baseline *T*_bio_ and baseline imaging parameters (GMV in Broca's or Wernicke's areas, MD of AC and EmC tracts, functional connectivity between the two brain regions).

## DISCUSSION

Our findings indicate a strong modulatory influence of testosterone on language‐specific gray and white matter structures as well as functional connectivity. Furthermore, they affirm that the neuronal organization of language‐related brain regions is subject to neuroplastic adaptation even in adulthood [Dehaene et al., 2010]. Similar effects of neuroplasticity have also been described for other cognitive functions, where taxi drivers or musicians showed increased GMV in specific brain regions [Zatorre et al., 2012] related to their professional expertise. Longitudinal studies further specified that such increases in GMV were already apparent after 7 days of training [Zatorre et al., 2012]. The brain regions identified in this work already showed testosterone‐dependent neuronal activation during synonym generation and match with the auditory ventral stream specifically involved in phonetic processing [DeWitt and Rauschecker, 2012]. In line with previous findings [Lombardo et al., 2012; Witte et al., 2010], the negative correlation between testosterone and GMV may therefore reflect an attenuation of language function in FtM subjects during treatment [Gooren and Giltay, 2008]. As increases in testosterone levels lead to masculinization of FtM transsexuals [Bao and Swaab, 2011], our observations link known sex differences in cognitive processing [Hollier et al., 2013; Wolf et al., 2000] to the particular influence of testosterone on gray matter structures. This is further complemented by results of increased volumes and cell packing density in Broca's and Wernicke's areas for women when compared to men [Harasty et al., 1997; Jacobs et al., 1993]. These findings may also be relevant for neuroplasticity of language, corroborating known sex differences between boys and girls regarding language impairment [Hollier et al., 2013] and stuttering [Boyle et al., 2011]. Furthermore, increased fetal testosterone levels have been associated with autism [Baron‐Cohen et al., 2015] and autistic traits [Auyeung et al., 2009]. This is particularly interesting as also FtM transsexuals show higher autistic traits [Jones et al., 2012] and autism subjects may in turn exhibit a poor language function [Lombardo et al., 2015].

The abovementioned learning‐related effects of neuroplasticity have not only been observed within gray matter but also for white matter fiber tracts [Zatorre et al., 2012]. Independent studies demonstrated that specific training of rats increased white matter FA with accompanied decreases in mean and radial diffusivities. Histological assessment confirmed increases in myelin staining in both cases [Blumenfeld‐Katzir et al., 2011; Sampaio‐Baptista et al., 2013]. In contrast to our initial hypothesis, our negative association between testosterone and EmC mean diffusivity therefore suggests a strengthening of this specific language pathway. Together with our first finding, it seems that testosterone actually exhibits an opposing influence on gray and white matter structures responsible for language processing. In line with this interpretation, longitudinal changes in GMV were inversely related to changes in white matter volume and FA in healthy adolescents, potentially reflecting cortical synaptic pruning and enhancement of tract diameters, respectively [Giorgio et al., 2010]. Similarly, children trained in abacus calculations showed a negative association between fusiform gyrus volume and associated white matter FA, whereas control subjects exhibited a positive relationship [Li et al., 2013]. The beneficial effect of testosterone on white matter microstructure is further supported as it impacts myelin formation. Castration of mice resulted in demyelination, whereas subsequent testosterone administration led to re‐myelination of the corpus callosum [Patel et al., 2013]. Moreover, positive associations have been reported between testosterone and white matter FA in boys [Herting et al., 2012] and increased FA in FtM after hormone treatment [Rametti et al., 2012]. Finally, such increases in FA near Broca's area predicted success rates in learning novel grammar rules [Floel et al., 2009]. To sum up, our results indicate that testosterone‐induced adverse effects on GMV in Broca's and Wernicke's areas seem to be compensated by a potential enhancement of myelin formation in the EmC white matter language pathway.

Although the AF has traditionally been considered the dominant language pathway [Catani and Mesulam, 2008], being responsible for auditory‐motor integration [Saur et al., 2008] and language production, the EmC tract is specifically involved in higher order language function such as semantic processing and language comprehension [Saur et al., 2008], though it is often neglected in current research. Accordingly, lesion volumes in dorsal and ventral white matter pathways of aphasic stroke patients were negatively associated with repetition and comprehension performance, respectively [Kummerer et al., 2013]. Furthermore, language impairment in adolescents born preterm was related to volume reductions of the EmC pathway but not the AF [Northam et al., 2012].

The above findings are further complemented by the observed functional connectivity between Broca's and Wernicke's areas, showing an enhancement with increasing testosterone levels similar to structural connectivity. This relationship is supported by decreased superior temporal gyrus functional connectivity in women [Biswal et al., 2010], which increased after a single administration of testosterone [Schutter et al., 2005]. Accordingly, various reports linked higher functional connectivity to improved language skills. For instance, positive associations have been established between functional connectivity and reading abilities in native and second language [Zhang et al., 2014]. Conversely, language performance and functional connectivity are decreased in stuttering adults [Chang et al., 2011] and epilepsy patients [Vlooswijk et al., 2010]. Together with the first two results, it appears that testosterone exhibits complex effects on the language‐specific brain network. Consistent with the enhanced structural connection passing through the extreme capsule, also the corresponding functional connectivity between language regions was strengthened. On the other hand, decreases in local GMVs of Broca's and Wernicke's areas with increasing testosterone levels were demonstrated. Such a compensatory effect of brain connectivity is supported by a previous investigation, where several connections between language‐related areas were specifically activated to correct for auditory feedback perturbations [Tourville et al., 2008]. Hence, it appears that important regions and connections of the language network [Saur et al., 2010] are subject to testosterone‐specific adaptations, in which enhanced connectivity may compensate for local deteriorations.

It is worth mentioning that the actual changes in GMV, white matter mean diffusivity and functional connectivity were apparent in both positive and negative directions. This suggests that testosterone may exhibit a dose‐dependent influence on brain structure and function. Testosterone is converted to 17β‐estradiol and 5α‐dihydrotestosterone by the enzymes aromatase and 5α‐reductase, however, these metabolic processes are saturable [Lakshman et al., 2010]. Men homozygous for the A‐allele of the gene coding for aromatase showed higher conversion of testosterone to estradiol, hence, increased estradiol serum levels [Bayer et al., 2013]. Taking into account that the same subjects also exhibited greater hippocampal GMV [Bayer et al., 2013], it seems that testosterone and estradiol have opposite effects on human brain structure. This is corroborated by positive and negative GMV associations with estradiol and testosterone, respectively [Witte et al., 2010], as well as contrary influences of the two hormones on white matter maturation [Prayer et al., 1997]. Conversely, language performance at 4 years of age correlated negatively with testosterone and positively with estradiol obtained at infancy [Schaadt et al., 2015]. We further speculate that after saturation of aromatase‐related conversion to estradiol [Lakshman et al., 2010], the increased binding of testosterone to the androgen receptor may represent the dominant factor for the subsequent impact on brain structures. In line with this hypothesis, the androgen receptor gene modulated testosterone‐dependent gray matter decreases in men, with less CAG repeats representing higher transcriptional activity and being associated with stronger gray matter decreases [Paus et al., 2010]. In this context, the lack of significant results for the general effects of treatment (i.e., interaction effect group * time) is not surprising. Again, the opposite effects of testosterone and estradiol on gray and white matter brain structures [Bayer et al., 2013; Prayer et al., 1997; Witte et al., 2010] as well as language performance [Schaadt et al., 2015] would average out simple effects of time, which was observed in the current study. Furthermore, assessing general effects of treatment is less specific as any change accompanying the treatment may potentially yield a significant result.

As a limitation of this study, no cognitive tests on language function were available. Hence, the interpretation that testosterone affects language skills (via the demonstrated influence on brain structure and function) has to rely on previous reports showing that increased testosterone predicts smaller vocabulary in boys [Hollier et al., 2013] as well as language performance [Schaadt et al., 2015] and that testosterone administration in FtM transsexuals decreases verbal fluency skills [Gooren and Giltay, 2008; Van Goozen et al., 1995]. Similarly, we cannot exclude that a simple change from language‐related to physical activities due to testosterone administration may cause the observed changes. Though, no such inverse effects (i.e., GMV increases) were observed in primary or supplementary motor areas, which would indicate increased physical activities. The lack of significant correlations with baseline testosterone values as found in previous studies [Lombardo et al., 2012; Peper et al., 2015; Witte et al., 2010] may be explained by several factors such as sample size, inclusion of male subjects and vice versa the generally low testosterone values of women. Hence, it is possible that the effects observed here require larger absolute values (such as available in men) or large changes in testosterone.

In conclusion, the observed modulatory influence of testosterone on gray and white matter brain structures as well as functional connectivity links known sex differences in language performance to hormonal effects on associated brain regions. These neuroplastic adaptations however seemed to be opposed, with decreased GMVs in Broca's and Wernicke's areas but a beneficial impact on the corresponding functional connectivity and the white matter tract passing through the extreme capsule.
